# Red- and Near-Infrared-Excited Autofluorescence as a Marker for Acute Oxidative Stress in Skin Exposed to Cigarette Smoke Ex Vivo and In Vivo

**DOI:** 10.3390/antiox12051011

**Published:** 2023-04-27

**Authors:** Phuong Thao Tran, Parichat Tawornchat, Burkhard Kleuser, Silke B. Lohan, Johannes Schleusener, Martina C. Meinke, Maxim E. Darvin

**Affiliations:** 1 Charité—Universitätsmedizin Berlin, Corporate Member of Freie Universität Berlin and Humboldt-Universität zu Berlin, Department of Dermatology, Venereology and Allergology, Center of Experimental and Applied Cutaneous Physiology, Charitéplatz 1, 10117 Berlin, Germany; 2 Institute of Pharmacy, Department of Pharmacology, Freie Universität Berlin, Königin-Luise-Str. 2+4, 14195 Berlin, Germany; 3 Center of Excellence on Petrochemical and Materials Technology, Chulalongkorn University, Bangkok 10330, Thailand

**Keywords:** cigarette smoke, oxidative stress, Raman spectroscopy, NIR autofluorescence, red autofluorescence, skin fluorophores, metabolic imaging

## Abstract

Air pollution is increasing worldwide and skin is exposed to high levels of pollution daily, causing oxidative stress and other negative consequences. The methods used to determine oxidative stress in the skin are invasive and non-invasive label-free in vivo methods, which are severely limited. Here, a non-invasive and label-free method to determine the effect of cigarette smoke (CS) exposure on skin ex vivo (porcine) and in vivo (human) was established. The method is based on the measurement of significant CS-exposure-induced enhancement in red- and near-infrared (NIR)-excited autofluorescence (AF) intensities in the skin. To understand the origin of red- and NIR-excited skin AF, the skin was exposed to several doses of CS in a smoking chamber. UVA irradiation was used as a positive control of oxidative stress in the skin. The skin was measured with confocal Raman microspectroscopy before CS exposure, immediately after CS exposure, and after skin cleaning. CS exposure significantly increased the intensity of red- and NIR-excited skin AF in a dose-dependent manner in the epidermis, as confirmed by laser scanning microscopy AF imaging and fluorescence spectroscopy measurements. UVA irradiation enhanced the intensity of AF, but to a lower extent than CS exposure. We concluded that the increase in red- and NIR-excited AF intensities of the skin after CS exposure could clearly be related to the induction of oxidative stress in skin, where skin surface lipids are mainly oxidized.

## 1. Introduction

The increase in air pollution over the years has had a major effect on human health and the quality of life. In 2022, the World Health Organization reported that exposure to air pollution (e.g., polycyclic aromatic hydrocarbons, volatile organic compounds, oxides, particulate matter, and ozone) was estimated to cause over 7 million annual premature deaths worldwide [1]. The majority of deaths are caused by ischemic heart disease, stroke, and chronic obstructive pulmonary disease; in addition, air pollution has enormous effects on human skin [2,3].

Cigarette smoke (CS) is a pollutant that consists of particulate matter, polycyclic hydrocarbons, and thousands of other components [4]. Active and passive smokers are exposed to the fumes of cigarettes on a daily basis. CS induces oxidative stress and contributes to the development of premature skin aging and several inflammatory pathologies [5,6].

Skin—especially its superficial layer, the stratum corneum—acts as the most important defense barrier against environmental contaminants. Exposure to air pollutants can induce a harmful effect on the skin by increasing the concentration of reactive oxygen species (ROS). The excess of ROS disturbs the oxidant/antioxidant balance, resulting in oxidative stress. Oxidative stress induces severe alterations of lipids, DNA, proteins, antioxidants, etc., in the skin, leading to impairment of the skin barrier function and the skin’s protection ability. It can also lead to the development of premature skin aging and inflammatory or allergic conditions, such as contact dermatitis, atopic dermatitis, eczema, psoriasis, and acne, as well as skin cancer, which is the most serious result [7,8,9].

Currently, methods of evaluating air-pollution-induced skin damage are limited. Most of these methods are indirect and/or invasive (e.g., skin biopsies, blood sampling, tape-stripping, trans-epidermal water loss measurements, and epidemiological studies over a long time period) [10,11,12,13,14]. In addition, there are in vitro experiments (e.g., cytotoxicity, collagen metabolism, glutathione assays) [15,16,17], which might not always be representative for non-invasive in vivo skin studies [18,19,20].

To address the above challenges, a label-free optical method to investigate depth-dependently the effect of CS exposure on skin was applied. Red- and near-infrared (NIR)-excited autofluorescence (AF) intensities in skin were measured depth-dependently on excised porcine skin biopsies and on healthy human volunteers after dose-dependent CS exposure and UVA irradiation using a confocal Raman microscope. As control, compartments of CS alone were investigated and the skin was measured directly after exposure and after a cleaning procedure to remove particular matter and compounds on the skin surface, which could potentially influence AF intensity.

## 2. Materials and Methods

### 2.1. Preparation of Ex Vivo Porcine Skin Samples

Porcine ears were obtained from a local butcher, cleaned with cold water, and stored at 4 °C. The ears were used within 48 h after slaughtering. Experiments were approved by the veterinary office in Dahme–Spreewald, Germany, in accordance with Section 3, Article 17, paragraph 1, of Regulation (EC) No 1069/2009 of the European Parliament and Council dated 21 October 2009, which establishes health regulations for animal byproducts that are not intended for human consumption.

To perform the experiments, the hair on the porcine ear skin was trimmed with scissors without affecting the stratum corneum. Subsequently, the trimmed skin area was cut to ≈1 × 1 cm^2^ pieces. At least five pieces per ear were prepared for analysis.

### 2.2. Preparation for In Vivo Human Skin Study

Ten healthy Caucasian volunteers (five male and five female) aged from 20 to 36 years (mean 27.2 ± 4.5 years) were included in the study. Nine of the ten volunteers were nonsmokers. No skin care products were used on the volunteers’ inner forearms for at least 12 h before the experiments. At first, the volunteers acclimated for 10 min in the laboratory at set conditions (temperature +20 °C, relative humidity ≈40–60%). At least six positions on their inner forearms were used for measurements.

Before starting the experiment, the study design and possible risks were explained and the volunteers provided their informed written consent. This study was approved by the Ethics Committee of the Charité—Universitätsmedizin Berlin (EA1/291/21, DRKS00029235) and all procedures followed the Code of Ethics of the World Medical Association (Declaration of Helsinki).

### 2.3. Cigarettes

Research cigarettes (1R6F, University of Kentucky, Lexington, KY, USA) (ISTD = internal standard) [21] and commercially available cigarettes (Gauloises Blonde Bleu, Tarnowo Podgórne, Poland) were used in this study. The composition of the two kinds of cigarettes are comparable. The ingredients of the research cigarettes 1R6F (mg/cig) were tar (8.6 mg); nicotine (0.72 mg); and carbon monoxide (10.1 mg) [22]. The ingredients of Gauloises Blonde Bleu (mg/cig) are tar (10 mg); nicotine (0.8 mg); and carbon monoxide (10 mg) [23].

### 2.4. Cigarette Smoke Exposure on Skin

**Figure 1:**
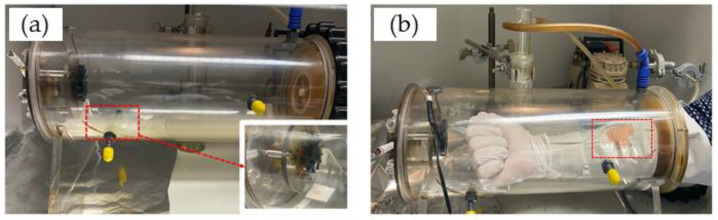
Exposure of a skin sample ex vivo (**a**) or human forearm skin in vivo (**b**) to cigarette smoke in a smoking chamber.

The porcine skin sample was placed in an exposure chamber that was designed by the Charité for exposure to CS generated by the combustion of Gauloises or research 1R6F cigarettes. For the first ex vivo measurements, Gauloises cigarettes were used. For repetitions and in vivo experiments, the 1R6F cigarettes were used. The smoking chamber was constructed by the Centrum Wissenschaftliche Werkstaetten (CWW) der Charité—Universitaetsmedizin Berlin. This chamber was used for ex vivo and in vivo studies, as illustrated in Figure 1. In a previous study, the reproducible CS exposure was measured and validated [6]. The chamber was connected to a pump, which represents the continuous aspiration of CS. The dose of CS exposed on the skin surface was controlled by the number of cigarettes and the pump time. For CS exposure on human skin in vivo, gloves protecting the skin of both hands and arms were used; only the measurement area of ≈3 × 4 cm^2^ was left unprotected and sealed with tapes (Figure 1b).

In the case of five cigarettes and one cigarette, the pump was turned on for 1 min after one cigarette or five cigarettes were simultaneously lighted. For ½ and ¼ cigarettes, the pump was turned on for 30 s and 15 s, respectively, after a cigarette was lighted. After the pump was turned off, all experimental samples were incubated in the chamber for a total of 5 min. After exposure, the skin was immediately investigated using confocal Raman microspectroscopy (CRM). Then, the skin piece was cleaned using distilled water (1 mL/cm^2^ skin) to remove any chemicals/particulate matter on the skin surface and was again investigated using CRM. Intact non-exposed skin was used as a negative control. UVA irradiation (2 minimal erythema dose (MED)) was used as positive control.

For each experiment, an absorbing filter paper (3.5 × 2.5 cm^2^) was inserted into the chamber next to the measurement area on the forearm or skin sample to investigate the nicotine concentration exposed on the skin surface. To extract the nicotine, the exposed filter paper was incubated in 10 mL of ethanol (Uvasol^®^ Ethanol 99.9%, Merck KGaA, Darmstadt, Germany). Then, the solution was measured by a UV spectrometer (Perkin Elmer, Inc., Waltham, MA, USA) to determine the nicotine content with the aid of a standard curve prepared from nicotine standard solutions, using the maximum wavelength of nicotine in ethanol at 262 nm and pure ethanol as a blank. The method was previously described in detail by Tran et al. [6].

### 2.5. Cigarette Smoke Exposed on a Glass Slide

An uncoated glass slide (R. Langenbrinck GmbH, Emmendingen, Germany) was put into the smoking chamber and exposed with five cigarettes. The smoking-induced particles deposited on the glass slide were further measured with CRM.

### 2.6. UVA Irradiation of Porcine Skin

To induce oxidative stress (positive control), skin samples were irradiated using an UVA–LED lamp at 365 ± 5 nm (Freiberg Instruments GmbH, Freiberg, Germany) for 106 min, while untreated skin was used as a negative control and subjected to CRM analyses. The applied UVA dose was 52 J/cm^2^, which equals 2 MED and is sufficient to induce oxidative stress in the skin [24].

### 2.7. Chemical Induced Oxidation of Porcine Skin

To induce chemical oxidation in excised porcine skin, the skin samples were each incubated with 150 µL/cm^2^ 2 mM hydrogen peroxide, according the protocol of Hergesell et al. [25], and 30% hydrogen peroxide for 30 min.

### 2.8. Red and NIR Excited Autofluorescence of Nicotine

One drop of pure nicotine (Caesar and Loretz GmbH, Hilden, Germany) was transferred to an uncoated glass slide. The Raman spectrum was recorded using CRM.

### 2.9. Confocal Raman Microspectroscopy (CRM)

For ex vivo and in vivo measurements, the Model 3510 skin composition analyzer (RiverD International B.V., Rotterdam, The Netherlands) was used. For the fingerprint region (FP: 400–2000 cm^−1^), the excitation wavelength was 785 nm, the exposure time 5 s, and the power 20 mW. For the high wavenumber region (HWN: 2000–4000 cm^−1^), the excitation wavelength was 671 nm, the exposure time was 1 s, and the power was 17 mW [26,27]. Raman spectra were recorded from the skin surface down to 40 µm at 2 µm increments. The utilized CRM was described in detail elsewhere [28]; it is widely used for the analysis of skin composition and drug penetration [29,30,31].

### 2.10. Confocal Laser Scanning Microscopy (LSM) and Fluorescence Microscopy

To investigate the effect of CS exposure on the skin surface, a confocal laser scanning microscope (LSM, VivaScope^®^ 1500, Multilaser, MAVIG, Munich, Germany) was used to capture the AF in skin. In this study, the laser diode of 785 nm was chosen. Before and after CS exposure, a laser power of 5 mW was used to compare recorded AF intensities.

For both samples, a drop of immersion oil (Crodamol STS, Croda Inc., Snaith, UK) was applied on the skin and fixated by a ring glass (adhesive window with crosshair, Lucid Vision Labs GmbH, Ilsfeld, Germany). For optimal optical contact, ultrasonic gel (Aquasonic 100, Parker laboratories Inc., Fairfield, CT, USA) was applied between the objective and the ring of the investigated sample.

To confirm the results of the LSM, tape strips of untreated and CS exposed skin were taken to measure the AF of the skin samples with fluorescence microscopy.

For the tape-stripping method, cyanoacrylate was applied on the porcine skin and covered with adhesive tapes (Tesa^®^, No. 5529, Beiersdorf AG, Hamburg, Germany). With a weighted rubber roller of 746 g, the area was rolled 10 times without additional pressure. After 20 min, the adhesive tapes were collected and cut into two 1 × 1 cm^2^ pieces per porcine ear. Every piece was solved in 2 mL ethanol (Uvasol^®^ Ethanol 99.9%, Merck KGaA, Darmstadt, Germany) that was added to the ultrasonic bath (Banderlin Sonorex Super RK 102H, Berlin, Germany) for 10 min at 35 kHz and, afterwards, centrifuged at 1.920× *g* for an additional ten minutes. The supernatant of the solution was pipetted, filled into 500 µL cuvettes, and measured by fluorescence spectroscopy (LS-55, PerkinElmer Inc., Waltham, MA, USA). The excitation wavelength was 785 nm. Absorbance was measured in the wavelength range of 820–900 nm at a rate of 100 nm per minute.

### 2.11. Data Analysis

#### 2.11.1. Comparison of Autofluorescence Intensities

Images measured by the LSM were analyzed with the ImageJ software (Wayne Rasband, National Institute of Health, Bethesda, MD, USA, Version 1.54b). To compare the AF intensity values, the brightness was determined.

#### 2.11.2. Determination of Depth-Dependent Autofluorescence Intensity

To quantify the AF intensity, the fluorescence background in the fingerprint Raman spectra at the position 1800 cm^−1^ was used due to the absence of superposition with Raman bands at this wavenumber. For the high wavenumber region, the AF intensity was determined at position 2600 cm^−1^ due to the absence of superposition with Raman bands.

The mean AF intensity was determined depending on the skin depth. The investigations were performed before CS exposure, right after CS exposure, and after an additional cleaning step with distilled water following CS exposure.

### 2.12. Statistical Analysis

The data are presented as mean ± standard error of the mean (SEM). For statistical analysis, IBM Statistical Package for the Social Sciences (SPSS) Statistics version 28 (IBM Corporation, Armonk, NY, USA) was applied. Normal distribution was tested using the Shapiro–Wilk test. To compare significant differences, the Kruskal–Wallis Test with the (Dunn–Bonferroni) post hoc test was used. The significance between the increase of AF intensity in excised porcine skin and in vivo human skin over the entire measurement was calculated with a generalized estimating equation (GEE). A *p*-value ≤ 0.05 was considered to be statistically significant.

## 3. Results

### 3.1. Cigarette Smoke Increases NIR- and Red-Excited Autofluorescence Intensity in Ex Vivo Porcine Skin

**Figure 2:**
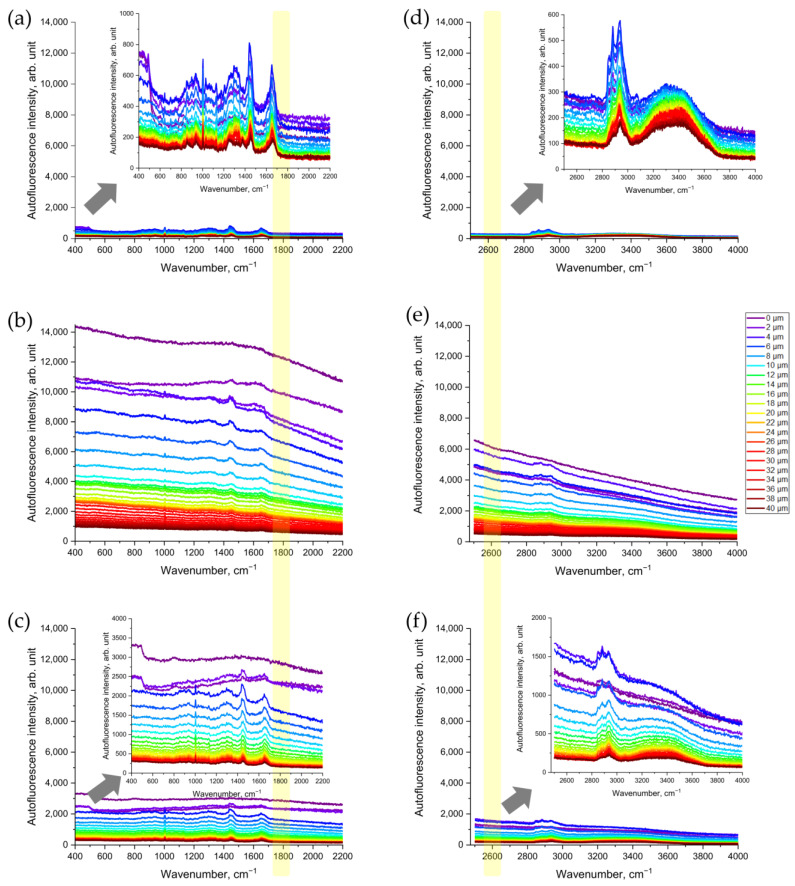
Representative Raman spectra of CS-exposed and non-exposed porcine skin at different depths (0–40 µm) excited in NIR with a wavelength of 785 nm (**a**–**c**) and in red with a wavelength of 671 nm (**d**–**f**) spectral ranges: (**a**,**d**) control skin before CS exposure; (**b**,**e**) uncleaned skin after CS exposure; (**c**,**f**) cleaned skin after CS exposure. The arrows point to the inserts in (**a**,**c**,**d**,**f**) that show the zoomed spectra, for clarity. The yellow color shows the area where the AF intensity was analyzed where no Raman bands can interfere.

First, we assessed whether CS exposure would change the Raman spectra of porcine skin that was being excited in the NIR range (Figure 2a–c). As shown in Figure 2b, the NIR excited AF intensity in the representative Raman spectrum of CS-exposed porcine skin increased dramatically—about 10-fold compared to the non-exposed skin (Figure 2a). Skin surface cleaning results in an obvious reduction in AF intensity (Figure 2c). However, even after removing the CS residue on the skin surface, the AF intensity was still approximately three times higher, compared to that of the non-exposed skin serving as control (Figure 2c).

Next, we observed the increase in AF intensity excited in the red spectral range (Figure 2d–f). Compared to the non-exposed skin (Figure 2d), CS exposure resulted in an obvious increase in red-excited AF intensity (Figure 2e). Again, after the cleaning of CS-exposed skin, the AF intensity remained approximately three times higher (Figure 2f) than that in the non-exposed skin serving as control (Figure 2d).

### 3.2. Dose-Dependent Increase in NIR- and Red-Excited Autofluorescence Intensity of the Skin due to Cigarette-Smoke Exposure

**Figure 3:**
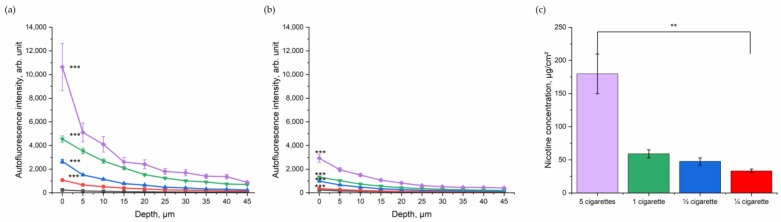
Depth profile of 785 nm NIR-excited AF intensity (mean ± SEM) of ex vivo porcine skin after dose-dependent exposure to CS (purple—five cigarettes, green—one cigarette, blue—½ cigarette, and red—¼ cigarette) immediately after CS exposure (**a**) and after cleaning (**b**), compared to control non-exposed skin (black). A general estimated equation was utilized; *** *p* ≤ 0.001 (CS-exposed vs. control skin). (**c**) Nicotine concentration (mean ± SEM) measured on a filter in the smoking chamber after exposure with five cigarettes in the smoking chamber (purple, 180 µg/cm^2)^; 1 cigarette in the smoking chamber (green, 59 µg/cm^2^); ½ cigarette in the smoking chamber (blue, 48 µg/cm^2^); and ¼ cigarette in the smoking chamber (red, 33 µg/cm^2^). Kruskal–Wallis test with Dunn–Bonferroni post hoc test. ** *p* ≤ 0.01.

After assessing the impact of CS in general in excised porcine skin, the dose-dependent relation was investigated. Porcine skin was exposed to ¼, ½, one, and five cigarettes in totals of five minutes (Figure 3). As shown in Figure 3a, the NIR-excited skin AF intensity increased with the number of burned cigarettes. Although the skin was cleaned, a dose-dependency between skin exhibiting NIR-excited AF intensity and the amount of burned cigarettes could be observed (Figure 3b). The exact amount of nicotine as a surrogate parameter for particle concentration on the skin was determined (Figure 3c) and significantly increased with the increasing number of burned cigarettes.

NIR-excited skin AF intensity was highest before cleaning the skin surface; however, even after cleaning there was a clear difference between the CS exposed and the control group in NIR-excited skin AF intensity. After cleaning, in the superficial depth (0–5 µm), the NIR-excited skin AF intensity was 2925 ± 344 arb. units after exposure with five cigarettes and, therefore, more than 10-fold higher than that of the control group (246 ± 21 arb. units). For one cigarette, the NIR-excited AF intensity after cleaning at the skin surface was 1331 ± 68 arb. units; for ½ cigarette, it was 1082 ± 91 arb. Units; and for ¼ cigarette, it was 355 ± 21 arb. units. The values decreased with increasing skin depth, as shown in Figure 3a,b.

### 3.3. Nicotine and Cigarette-Induced Residues Do Not Enhance NIR- and Red-Excited Skin Autofluorescence Intensity

**Figure 4:**
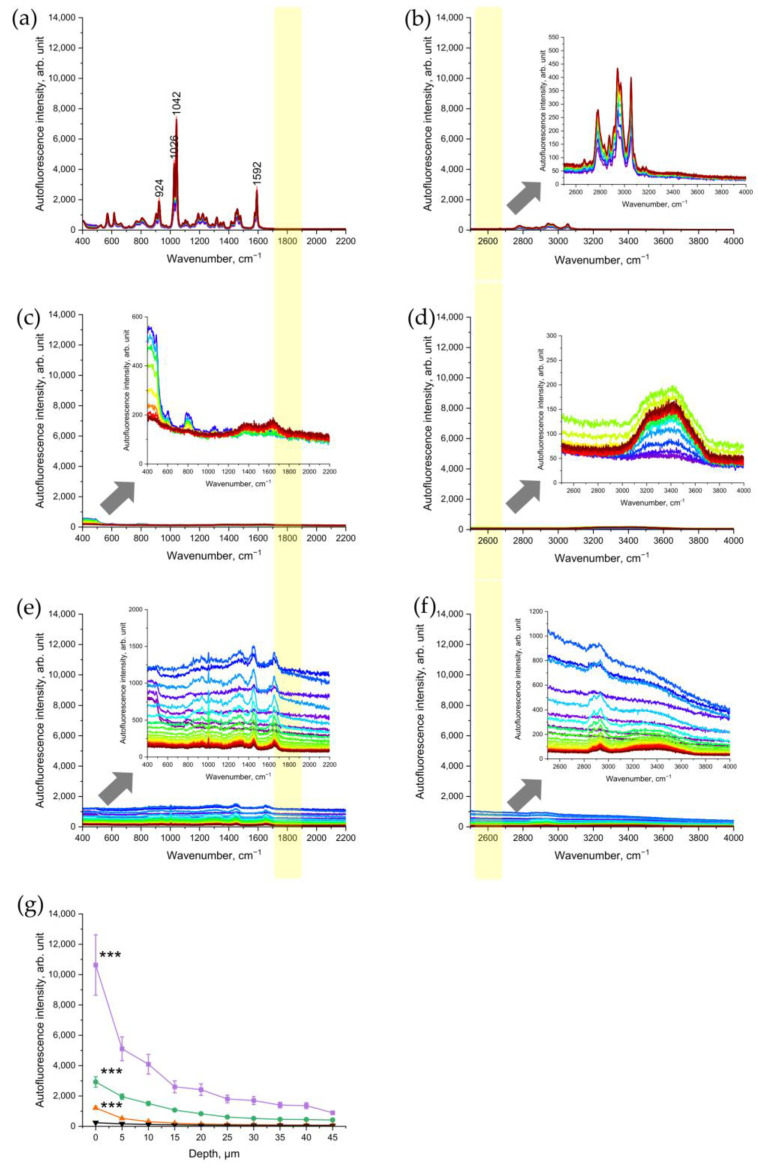
Representative Raman spectra of nicotine excited in 785 nm NIR (**a**) and in 671 nm red (**b**); spectral ranges of a glass slide after CS exposure excited in NIR (**c**) and in red (**d**); porcine skin excited in NIR (**e**) and in red (**f**) spectral ranges after exposure to UVA irradiation at 52 J/cm^2^ for 106 min (2 MED). The arrows point to the inserts in (**b**–**f**) that show the zoomed spectra for clarity. The yellow color shows the area where the AF intensity was analyzed where no Raman bands can interfere. (**g**) Comparison of NIR-excited AF intensity in porcine skin: five cigarettes immediately after 5 min of exposure (purple squares), five cigarettes after cleaning the skin sample (green circles), 2 UVA-MED with a 365 ± 5 nm LED for 106 min at 52 J/cm^2^ (red upward triangles), and control skin before CS exposure (black downward triangles). Graphs show mean ± SEM. GEE was applied; *** *p* ≤ 0.001 (CS-exposed vs. UVA-irradiated skin vs. control).

To verify that nicotine, as one of the main components of CS, is not the source of the NIR- and red-excited AF intensity of CS-exposed skin, pure nicotine was investigated with CRM. The results showed that nicotine is a Raman-active molecule, which does not generate any fluorescence signal under NIR- excitations (Figure 4a) or red excitations (Figure 4b). According to Baranska et al. [32], the peak at 1592 cm^−1^ could also refer to the pyridine ring, 1042 and 1026 and 924 cm^−1^ to C-C and C-N stretching vibrations of the alkaloid in the pyridine ring.

In addition, we measured the CS-exposed glass slide in order to check whether CS-related particles deposited on the skin surface are a source of NIR- and red-excited AF of CS-exposed skin. The results established that the CS-exposed glass slide did not show any significant increase in the NIR-excited (Figure 4c) and red-excited (Figure 4d) fluorescence intensity.

### 3.4. UVA Irradiation as a Positive Control of Oxidative Stress

As a positive control of oxidative stress, the changes in NIR-excited and red-excited AF intensity were analyzed in UVA-irradiated porcine skin. The results showed that UVA irradiation of the skin led to a significant depth-dependent increase in NIR- and red-excited AF intensity (Figure 4d,e). This result demonstrates that UVA-induced oxidative stress causes an increase in NIR-excited skin’s AF intensity. In comparison, at the skin surface (0 µm), the NIR-excited AF intensity in CS-exposed skin was approximately ten times higher than after UVA irradiation at 2 MED (Figure 4g). After cleaning the CS-exposed porcine skin, the AF intensity remained approximately two times higher than in UVA-irradiated skin. In the Appendix, the AF intensity of 2 UVA–MED is presented and compared with the samples of cleaned skin, for an overview. Here, the effect of 2 UVA–MED is comparable to that of one cigarette (Figure S2).

### 3.5. Chemically-Induced Oxidative Stress

In Figure S3, there is no significant difference in AF intensity between the control group and chemically-induced oxidative stress.

### 3.6. In Vivo Skin Measurements

In order to evaluate the effect of CS exposure on human skin in vivo, the ex vivo method was transferred to an in vivo setting. The lower forearm of each of the ten healthy human volunteers was exposed to five cigarettes within 5 min in the smoking chamber (Figure 1b). The NIR-excited AF intensity was measured before and after CS exposure and after cleaning the CS residue from the skin surface.

**Figure 5:**
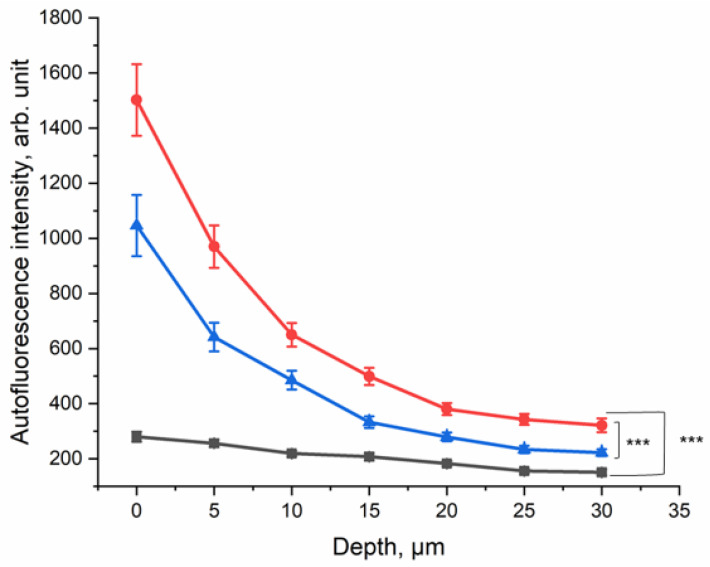
Depth profile of 785 nm NIR-excited AF intensity of CS-exposed human skin in vivo (*n* = 10) before CS exposure (black squares), after CS exposure to five cigarettes within 5 min (red circles), and after cleaning CS-exposed skin (blue triangles). The average nicotine concentration on the filter paper was 45.5 ± 9.3 µg/cm^2^. In depths of 0–30 µm, the difference between CS-exposed and non-exposed skin was significant (GEE, *** *p* ≤ 0.001).

The results are presented in Figure 5; they show that at the surface, the NIR-excited AF intensity was approximately five-fold higher in the skin of the CS-exposed group, compared to the non-exposed control area. Even after cleaning the skin after CS exposure, an approximately three-fold increase in NIR-excited AF intensity was measured in human skin in vivo. This result shows that CS exposure leads to an increase in NIR-excited AF intensity in human skin in vivo. NIR-excited Raman spectra of the human skin in vivo are shown in the Supplementary Materials (Figure S1a–c).

Red-excited Raman spectra of the human skin in vivo are shown in the Supplementary Materials (Figure S1d–f). A similar depth-dependent increase in the red-excited AF intensity of human skin in vivo was observed.

### 3.7. LSM Imaging Confirms an Enhancement of NIR-Excited Skin AF after Cigarette-Smoke Exposure

**Figure 6:**
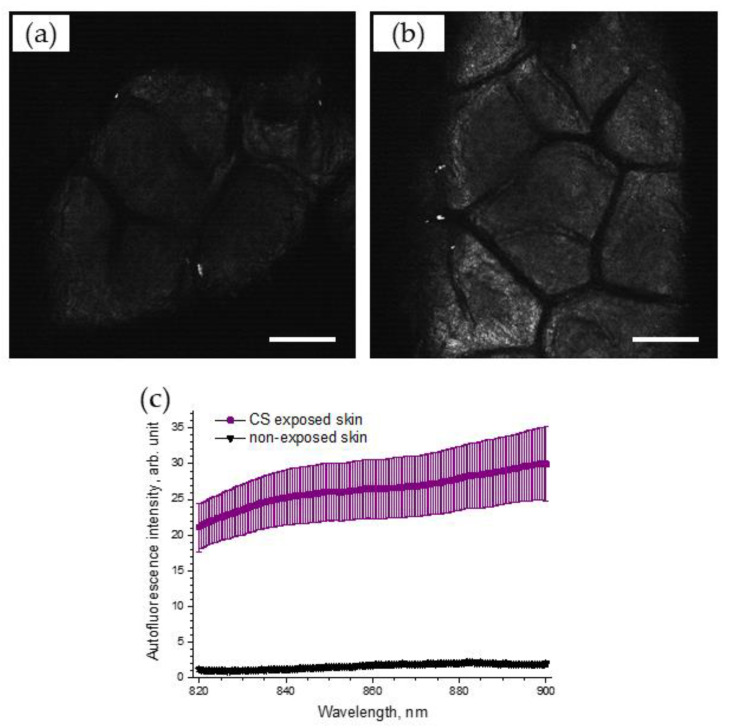
Exemplary 785 nm NIR-excited AF LSM images of the superficial stratum corneum of porcine skin before (**a**) and after (**b**) CS exposure to one cigarette within 5 min in the exposure chamber. Scale bar: 100 µm. (**c**) AF spectrum (mean ± SEM) of CS-exposed (five cigarettes within 5 min) porcine skin (purple) and non-exposed control skin (black), *n* = 6.

To support the obtained results, the AF intensities on the skin surface before and after CS exposure were recorded, using LSM. Figure 6 illustrates selected NIR-excited AF images of porcine skin before (Figure 6a) and after CS exposure (Figure 6b), recorded using LSM (785 nm). A strong, almost two-fold, increase in AF intensity, calculated as image brightness, could be observed in the LSM images of CS-exposed skin (4.0 arb. units), compared to that of non-exposed skin (1.9 arb. units).

The spectrum of 785 nm NIR-excited porcine skin AF is shown in Figure 6c for CS-exposed and non-exposed skin. The enhanced NIR-excited AF intensity in CS-exposed skin is obvious, compared to that of non-exposed skin (the average increase was 17.4 ± 3.6 times in the spectral range of 820–900 nm).

## 4. Discussion

The increase in air pollution not only affects the health of our lungs, but also our skin. So far, insufficient non-invasive methods are available to measure the effect of pollution, especially CS exposure during a short time period. We chose CS because it is among the most toxic environmental pollutants that include particulate matter, such as polycyclic hydrocarbons [4]. The nicotine concentration in the smoking chamber with five cigarettes for 5 min is comparable to spending 8 h in a bar where smoking is allowed [33]. Therefore, to emulate the real-life CS-exposure conditions, we changed the CS exposure dose from ¼ of a cigarette to five cigarettes within 5 min in the smoking chamber.

In this study, the non-invasive and label-free CRM method was used. It is a powerful technique to determine depth profiles of skin components and barrier-function-related parameters ex vivo and in vivo. Fluorescence background is always present in the Raman spectra and has an influence on the signal-to-noise ratio [34]. Usually, it is removed to analyze the Raman bands in a proper way. A short acquisition time significantly minimizes the effect of AF photobleaching [35] and allows AF intensity measurements to be very precise.

As shown in Figure 2a, at an NIR excitation of 785 nm, non-exposed porcine skin emits only a tiny amount of AF. The same is observed at red excitation of 671 nm (Figure 2d). After CS exposure, the NIR- and red-excited AF intensities of porcine skin ex vivo (Figure 2b,e) and human skin in vivo (Figure 5 and Figure S1) increased substantially. For NIR excitation, this was confirmed by LSM AF imaging (Figure 6a,b) and by measuring the spectrum of skin AF (Figure 6c) at the superficial depth for CS-exposed and non-exposed skin. The sensitivity of CRM for AF measurements is, however, much higher than that of the LSM imaging that was used.

In this study, our hypothesis was that CS exposure leads to an increase in NIR- and red-excited AF intensity of the skin through the induction of oxidative stress. In the NIR-excited fingerprint region and the red-excited high wavenumber region, there is no interference in the skin between AF and Raman bands at 1800 and 2600 cm^−1^, respectively. Therefore, these wavenumber positions were used to further calculate the NIR- and red-excited AF intensity of the skin. Analysis of skin Raman spectra after CS exposure did not reveal the appearance of new components that could help to identify the source of oxidative-stress products. Both ex vivo and in vivo experiments showed that the increase in NIR- and red-excited skin AF intensities was observed mainly in the stratum corneum up to the depth of 25 µm with highest intensity at the surface. The effect on the deeper epidermis was in agreement with the method used by Grether-Beck et al. [20], where the melanin located in the basal layer was stimulated by diesel-exhaust-particle exposure in in vivo skin.

To confirm the hypothesis that oxidative stress leads to an increase in NIR- and red-excited AF intensities in skin, the source of AF was investigated. As a negative control, no evidence was found that nicotine or the CS residue itself led to a significant increase in NIR- and red-excited skin AF intensities (Figure 4a–d). No correlation between the NIR- and red-excited AF intensities and the CS dose represented by nicotine concentration could be found (Figure 3a,b). As a positive control, we used UVA irradiation, which is known to induce oxidative stress in excised skin through the generation of ROS [36,37]. The significant increase in NIR- and red-excited AF intensities was measured in excised porcine skin after UVA irradiation at UVA–MED (Figure 4e,f). This might indicate that oxidative stress is not only induced by UVA irradiation but also by CS exposure, which increases NIR- and red-excited AF intensities in the skin.

After cleaning the ex vivo and in vivo skin following the CS exposure, an increased NIR- and red-excited AF intensity could still be measured depth-dependently and compared to non-exposed skin with a most pronounced change in the superficial layer of stratum corneum depth (Figure 3b and Figure 5). Skin cleaning removes the CS particulate residues located on the skin surface but, as shown in Figure 4a–d, such residues are not a source of fluorescence. Thus, we concluded that oxidized skin surface lipids are mainly responsible for the NIR- and red-excited AF intensities in the CS-exposed skin.

Skin AF was previously investigated in smokers, due to an increase in the advanced glycation end product concentration that serves as a biomarker for aging and is a contributing factor for degenerative diseases [38]. Endogenous fluorophores, such as porphyrins, that are allocated on skin [39] are known to emit AF at 785 nm [35]. As porphyrins play a key role in metabolic processes [40], oxidative stress induced by CS could increase their AF. Moreover, the oxidized lipids, proteins, and amino acids can serve as molecular sources of NIR- and red-excited AF in the skin [41]. It should be noted that in the skin, the number of fluorophores absorbing light in the red and NIR ranges is strongly limited, so that reabsorption is negligible and the depth-resolved detection of red and NIR quanta is determined very accurately [42].

Chemically-induced oxidation through hydrogen peroxide did not increase AF intensity, shown in Figure S3. Hydrogen peroxide that induces intracellular stress in skin was used as a positive control in the DCFH assays of Hergesell et al. [25]. Our method could not detect an increase in AF intensity in red and NIR spectral regions when oxidative stress was induced by hydrogen peroxide, compared to that of CS exposure and UV irradiation. One possible explanation could be the oxidation of fluorophores due to hydrogen peroxide, as was stated for AF induced by UV radiation combined with hydrogen peroxide [43] and in a study using microspectrofluorometry to detect AF emission from human leukemic living cells under oxidative stress [44]. Thus, not all intracellular stress can be detected by the presented red- and NIR-excited AF method.

It was previously shown that CS induces oxidative stress by 4-hydroxynoenal (4-HNE), a lipid peroxidation marker and a second messenger for oxidative stress [45], and an increase in pro-inflammatory interleukins [46,47] and via ROS [6]. Different pathways can lead to the development of oxidative stress in skin. In previous studies, ROS production due to UVA irradiation and CS exposure was investigated. Tran et al. [6] concluded that UVA irradiation with less than 1/3 MED induces more ROS in the skin than the exposure to five cigarettes. In contrast, the obtained results show that the effect on the skin is more pronounced by CS exposure than by UVA irradiation of 2 UVA–MED (Figure 4f). Therefore, CS exposure could induce effects other than the formation of ROS, because it leads to stronger enhancement of NIR- and red-excited AF intensities in the skin than UVA.

In in vivo human skin, the increase in NIR- and red-excited AF intensities was shown after CS exposure; in addition, even after cleaning the forearm, the increase was significant compared to the non-exposed control. The AF intensity after CS exposure in excised porcine skin was higher than in in vivo human skin, not only because of the higher CS concentration in the chamber but also because of the higher antioxidant status and, therefore, better protection in in vivo human skin [48,49]. Skin components in darker skin types emit higher NIR- and red-excited AF due to the higher melanin content [50]. The AF of melanin in the NIR at 785 nm was investigated by Huang et al. [51] and Han et al. [52] and in NIR at 785 nm and red at 671 nm by Yakimov et al. [50]. Here, a higher melanin content was related to a higher AF in the NIR- and red-excitation regions. This result shows that this method has limitations in measuring stratum spinosum and stratum basal epidermal layers of dark skin (skin types ≥ IV), as melanin interferes with the NIR- and red-excited AF induced by oxidative stress. The limitations are expected to be less-pronounced in the stratum corneum and stratum granulosum layers of dark skin. Porcine epidermis contains a much lower melanin concentration than human epidermis, enabling clear investigations of NIR- and red-excited AF without the superposition with melanin-excited AF [53]. For precise measurements, additional stress should be avoided, as short-term sun exposure could lead to an increase in NIR- and red-excited AF intensities. In the present in vivo human study, melanin did not interfere with the AF signal because the volunteers had skin types I–III, meaning that their melanin content in the epidermis was sufficiently low.

Nonetheless, we provide an alternative fast method to investigate the effect of CS on skin, because it is label-free and non-invasive. In addition, the effect of CS on the skin can be seen immediately by measuring skin AF, compared to the patch test of Grether-Beck et al. [20], where the results were only visible after nine days. Here, topical application of diesel exhaust particles led to an increase in skin pigmentation, due to induction of melanogenesis.

## 5. Conclusions

The results of the present work show that skin exposure to the model pollutant cigarette smoke (CS) led to the development of dose-dependent oxidative stress in the epidermis. Oxidative stress was detected non-invasively by a significant increase in 785 nm NIR-excited and 671 nm red-excited autofluorescence (AF) of the skin ex vivo and in vivo by analysis of depth-resolved Raman spectra. The intensity of the NIR- and red-excited AF was determined at positions of 1800 and 2600 cm^−1^, respectively, which did not contain Raman bands. Our findings demonstrate that the origin of AF of CS-exposed skin is not related to nicotine and other CS-induced residues in the superficial stratum corneum, but is related to induced oxidative stress, in which skin surface lipids are mainly involved. This was confirmed by the fact that cleaning CS-exposed skin resulted in a reduction in AF intensity, compared to uncleaned CS-exposed skin, but the AF intensity was still significantly higher than that of non-exposed skin. An increase in NIR-excited skin AF was also confirmed by laser scanning microscopy AF imaging and fluorescence spectroscopy. Thus, the novel label-free, non-invasive method for assessing oxidative stress in the skin due to CS exposure, based on the measurement of increased skin AF intensity, was presented. With this method, it will be possible in the future to assess the protective effects of anti-pollution skin care products.

## Data Availability

All data are contained within the article and the Supplementary Materials.

## Supplementary Materials

The following supporting information can be downloaded at: https://www.mdpi.com/article/10.3390/antiox12051011/s1, Figure S1: Representative Raman spectra of CS-exposed (five cigarettes within five minutes) and non-exposed human forearm skin in vivo at different depths (0–40 µm) excited in NIR (785 nm) (a–c) and in red (d–f) spectral ranges. (a,d) Control skin before CS exposure; (b,e) uncleaned skin after CS exposure; (c,f) cleaned skin after CS exposure. The inserts in (a,c,d,f) show the zoomed spectra for clarity. Figure S2: Depth profile of 785 nm NIR-excited AF intensity (mean ± SEM) of ex vivo porcine skin after dose-dependent exposure to CS (purple—five cigarettes; green—one cigarette; blue—½ cigarette; and red—¼ cigarette. Orange—2 UVA-MED) immediately after CS exposure and after cleaning the skin probes. Figure S3: Depth profile of 785 nm NIR-excited AF intensity (mean ± SEM) of ex vivo porcine skin after 30 min H_2_O_2_ incubation (red circles—2 mM H_2_O_2_; blue triangles—30% H_2_O_2_), compared to control excised porcine skin (black squares).
