# Supplementary material for: Red- and Near-Infrared-Excited Autofluorescence as a Marker for Acute Oxidative Stress in Skin Exposed to Cigarette Smoke Ex Vivo and In Vivo
Source: Antioxidants (Basel). 2023 Apr 27;12(5):1011. doi: 10.3390/antiox12051011 (PMC10215244; doi:10.3390/antiox12051011)
Supplement: Supplementary file 1 [file antioxidants-12-01011-s001.zip › antioxidants-2331199-supplementary.pptx]

## Slide 1
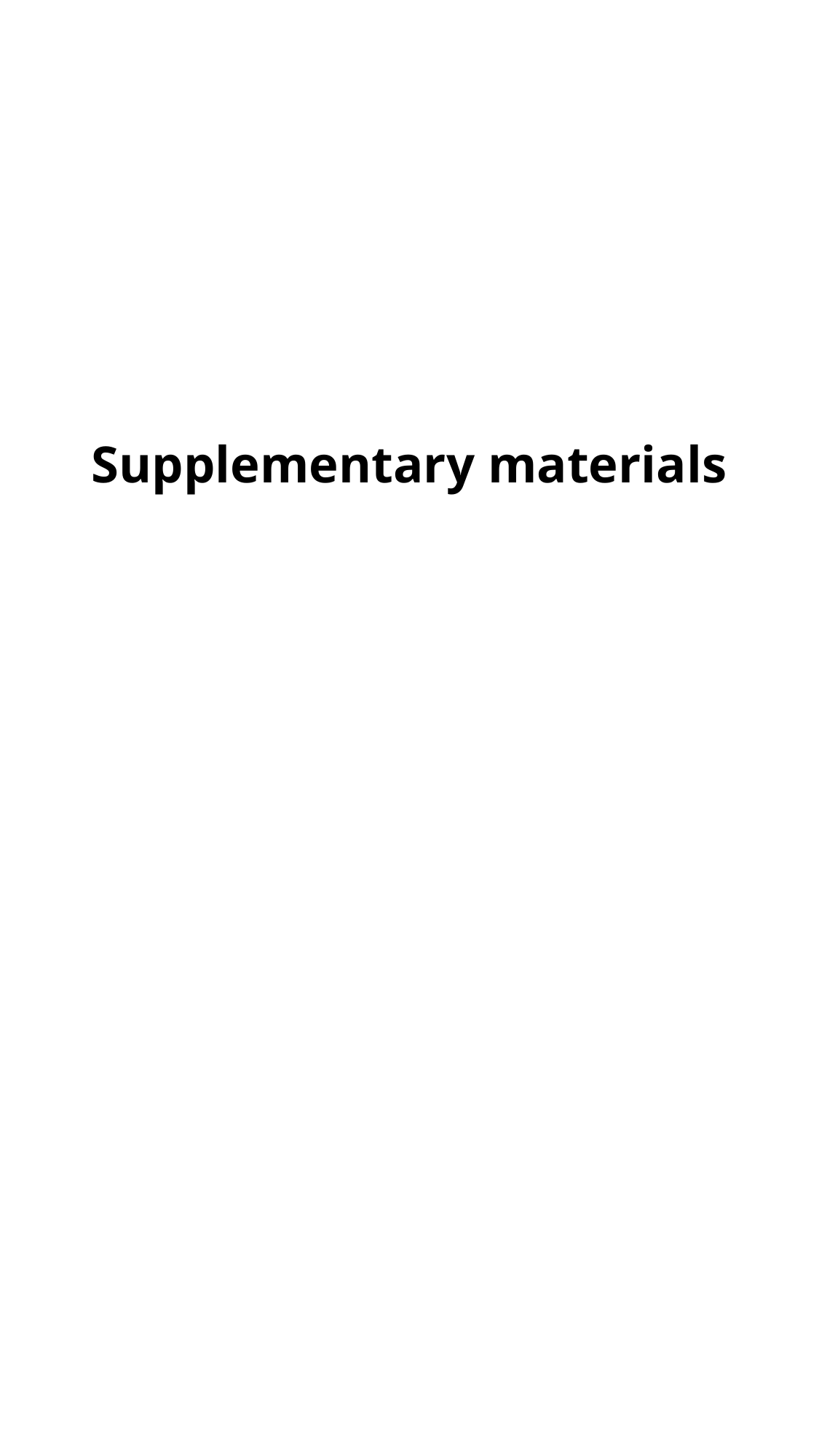

# Supplementary materials

## Slide 2
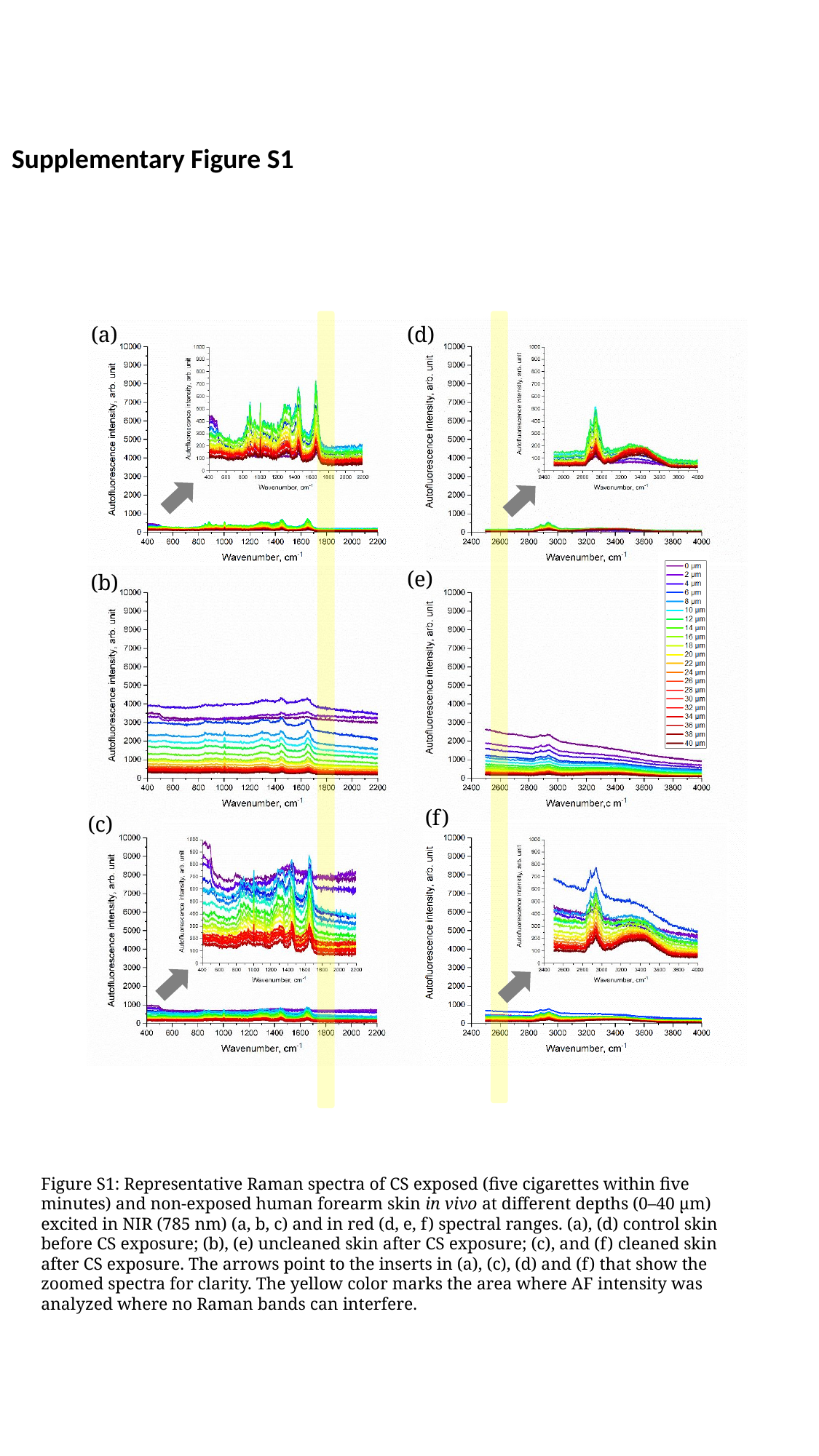

Supplementary Figure S1
(a)
(d)
(e)
(b)
(f)
(c)
Figure S1: Representative Raman spectra of CS exposed (five cigarettes within five minutes) and non-exposed human forearm skin in vivo at different depths (0–40 µm) excited in NIR (785 nm) (a, b, c) and in red (d, e, f) spectral ranges. (a), (d) control skin before CS exposure; (b), (e) uncleaned skin after CS exposure; (c), and (f) cleaned skin after CS exposure. The arrows point to the inserts in (a), (c), (d) and (f) that show the zoomed spectra for clarity. The yellow color marks the area where AF intensity was analyzed where no Raman bands can interfere.

## Slide 3
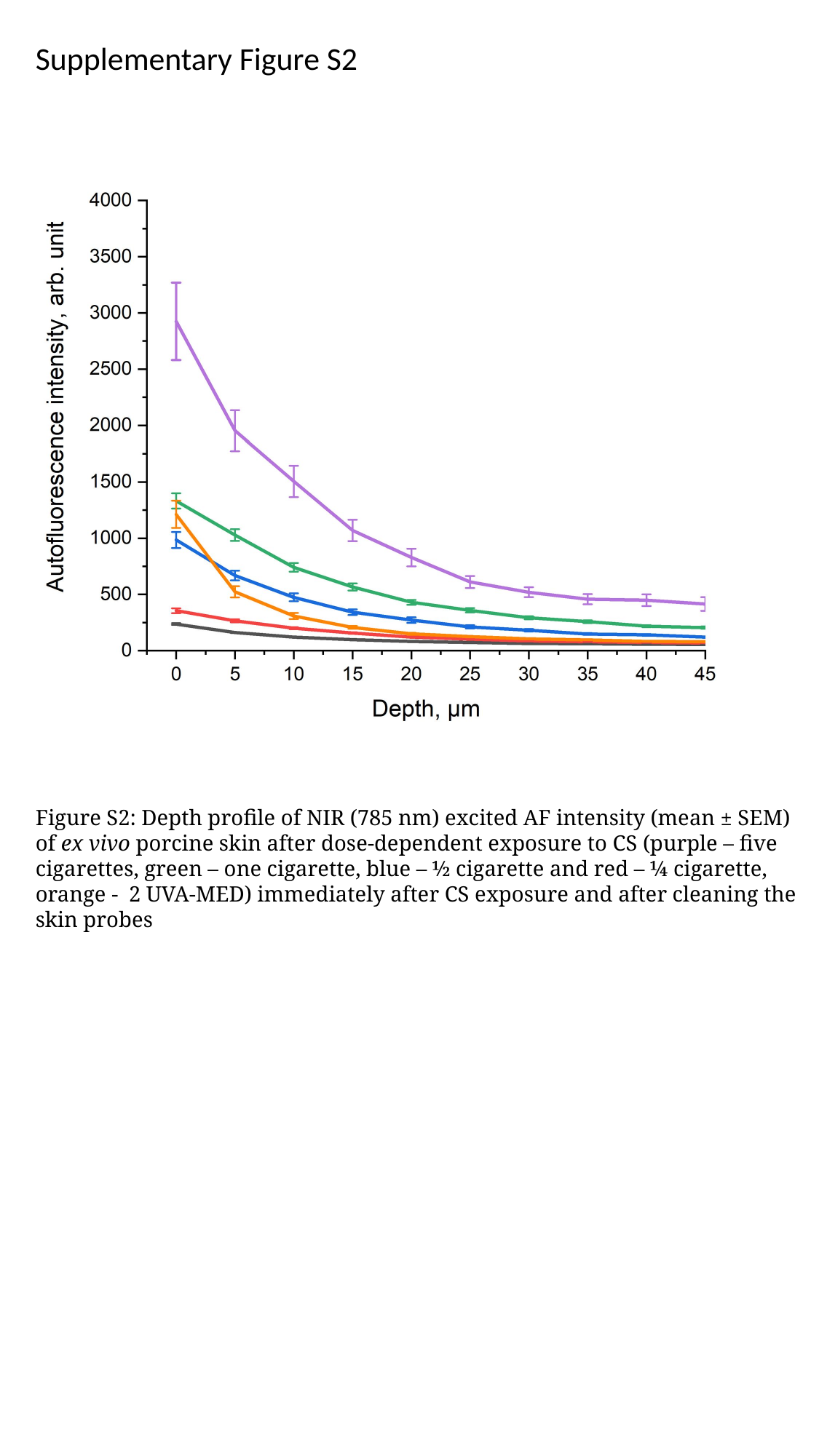

Supplementary Figure S2
Figure S2: Depth profile of NIR (785 nm) excited AF intensity (mean ± SEM) of ex vivo porcine skin after dose-dependent exposure to CS (purple – five cigarettes, green – one cigarette, blue – ½ cigarette and red – ¼ cigarette, orange - 2 UVA-MED) immediately after CS exposure and after cleaning the skin probes

## Slide 4
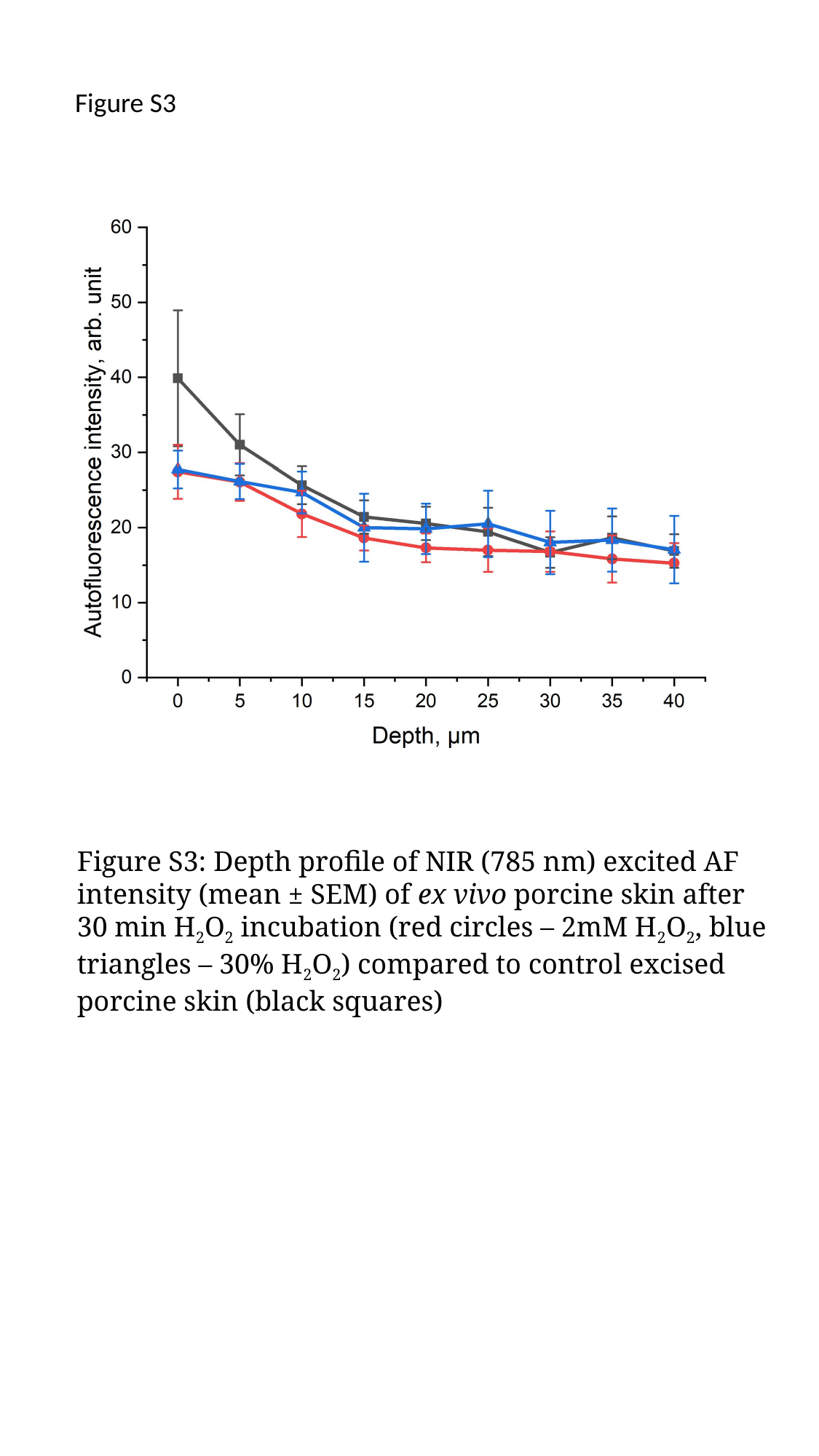

Figure S3
Figure S3: Depth profile of NIR (785 nm) excited AF intensity (mean ± SEM) of ex vivo porcine skin after 30 min H2O2 incubation (red circles – 2mM H2O2, blue triangles – 30% H2O2) compared to control excised porcine skin (black squares)
